# Quantifying the effect of air gap, depth, and range shifter thickness on TPS dosimetric accuracy in superficial PBS proton therapy

**DOI:** 10.1002/acm2.12241

**Published:** 2017-12-14

**Authors:** Robert J. Shirey, Hsinshun Terry Wu

**Affiliations:** ^1^ Radiation Oncology Aurora BayCare Medical Center Green Bay WI USA; ^2^ Radiation Oncology Willis‐Knighton Cancer Center Shreveport LA USA

**Keywords:** dose error, dosimetric accuracy, Monte Carlo, pencil beam, proton therapy, superficial, treatment planning

## Abstract

This study quantifies the dosimetric accuracy of a commercial treatment planning system as functions of treatment depth, air gap, and range shifter thickness for superficial pencil beam scanning proton therapy treatments. The RayStation 6 pencil beam and Monte Carlo dose engines were each used to calculate the dose distributions for a single treatment plan with varying range shifter air gaps. Central axis dose values extracted from each of the calculated plans were compared to dose values measured with a calibrated PTW Markus chamber at various depths in RW3 solid water. Dose was measured at 12 depths, ranging from the surface to 5 cm, for each of the 18 different air gaps, which ranged from 0.5 to 28 cm. TPS dosimetric accuracy, defined as the ratio of calculated dose relative to the measured dose, was plotted as functions of depth and air gap for the pencil beam and Monte Carlo dose algorithms. The accuracy of the TPS pencil beam dose algorithm was found to be clinically unacceptable at depths shallower than 3 cm with air gaps wider than 10 cm, and increased range shifter thickness only added to the dosimetric inaccuracy of the pencil beam algorithm. Each configuration calculated with Monte Carlo was determined to be clinically acceptable. Further comparisons of the Monte Carlo dose algorithm to the measured spread‐out Bragg Peaks of multiple fields used during machine commissioning verified the dosimetric accuracy of Monte Carlo in a variety of beam energies and field sizes. Discrepancies between measured and TPS calculated dose values can mainly be attributed to the ability (or lack thereof) of the TPS pencil beam dose algorithm to properly model secondary proton scatter generated in the range shifter.

## 1 INTRODUCTION

Willis‐Knighton Cancer Center is equipped with the ProteusONE system (IBA, Louvain‐La‐Neuve, Belgium) – a compact gantry pencil beam scanning proton therapy system with intensity modulated proton therapy (IMPT) capability and on‐board cone‐beam CT (CBCT). Treatment plans are created with the RayStation 6 treatment planning system (TPS), exported to MOSAIQ (Elekta, Sunnyvale, CA, USA), and treated on the ProteusONE. To date, WKCC has treated over 350 patients with brain, head & neck, thoracic (including breast, chest wall, lung, and spine), abdominal and pelvic tumors since September 2014.

Numerous publications have discussed, in varying degrees of detail, that pencil beam dose algorithms do not accurately model superficial dose distributions of range‐shifted proton pencil beam scanning (PBS) treatments.^1^, ^2^, ^3^ For example, the RayStation 5 User Manual specifically notes the dosimetric shortcomings of the RayStation pencil beam algorithm. “WARNING! Absolute dose accuracy for proton PBS and Line Scanning with range shifters. In the RayStation 5 PBS dose engine validation, a few deviations from the dose accuracy requirements were noted for doses at shallow depths (<3 cm) in water when a range shifter was used. Do not use the device in these situations.”^4^ RayStation updated the language in the RayStation 6 User Manual to explain the reasoning for the inaccurate dose calculation and suggests the use of the Monte Carlo dose engine to more accurately calculate dose in such situations.^5^

One of the main benefits of proton therapy is the ability to control the distal range of the treatment field by taking advantage of the Bragg Peak. This allows for the treatment of target volumes located proximal to normal tissue or organs at risk with little dosimetric detriment to the non‐target volumes.^6^ When target volumes are located relatively deep in the patient, the accuracy of the TPS is sufficient.^1^, ^3^ Targets such as chest wall, however, can have a significant portion of the target volume located a depths shallower than 3 cm. At such shallow depths, the minimum beam energy has a range greater than the target depth. A range shifter placed in the beamline sufficiently reduces beam energy such that full dose modulation is achievable at the patient surface. The ProteusONE is capable of producing a minimum beam energy of 70 MeV, which has a range in water of approximately 4.1 cm.^7^ WKCC commissioned a 3.5 cm physical thickness (4.1 cm water‐equivalent thickness) Lexan range shifter to treat shallow target volumes with the ProteusONE. Other proton therapy systems with minimum beam energies of 100 MeV would require a range shifter with approximately 7.5 cm water‐equivalent thickness.^7^ As noted above, the use of range shifters for shallow treatments can be problematic for a TPS using a pencil beam dose algorithm.

Though most commercially available proton TPS – including Pinnacle,^8^ XiO,^9^ Eclipse,^10^ and RayStation^4^ – use pencil beam dose algorithms, no published studies could be found which quantify the functional dependence of TPS dosimetric accuracy on depth or air gap. A selection of publications have quantified TPS accuracy for multiple depths with a fixed air gap,^11^ and other works have generally noted that a pencil beam algorithm breaks down with large air gaps and shallow depths.^2^, ^4^, ^5^, ^12^ This study, for the first time, systematically quantifies the dosimetric accuracy of a proton pencil beam dose algorithm as a function of range shifter air gap and treatment depth for superficial proton PBS treatments. Moreover, this study performed an identical analysis using the RayStation 6 Monte Carlo proton dose engine to determine the improvement in dosimetric accuracy one may expect when using Monte Carlo. Finally, a smaller subset of this study performed similar measurements with a thicker range shifter to identify the relationship between pencil beam TPS accuracy and range shifter thickness. This data was then tested against patient treatment plans to confirm its applicability to the clinical treatment environment. To further confirm the dosimetric accuracy of the RayStation 6 Monte Carlo dose algorithm at beam energies and field sizes other than those described above, MC‐calculated spread‐out Bragg Peak (SOBP) profiles were compared to measured SOBP's of a variety of fields used during machine commissioning.

## 2 METHODS AND MATERIALS

### 2.A TPS setup

A single treatment plan, from which all TPS computations were calculated, was created in RayStation 6. A CT dataset measuring 40 × 40 × 40 cm^3^ with 1 mm resolution was computer‐generated using Python (Python Software Foundation, Wilmington, DE, USA) and imported into the TPS. The material properties of the entire dataset were manually overridden with the physical density and elemental composition of RW3.

A single beam was created in an A‐P configuration with a 3.5 cm thick (4.1 cm WET) Lexan range shifter (RS) in the beam line and isocenter placement 13.45 cm below the phantom surface. The isocenter placement allowed for a minimum air gap of 0.5 cm and a maximum air gap of 28 cm with the range shifter in its most extended and retracted positions, respectively. Using a 1 × 1 × 1 mm^3^ dose grid and a target volume measuring 8 × 8 cm^2^ in the coronal plane and extending 6 cm deep from the phantom surface, the treatment plan was optimized to deliver a uniform dose to the target. The optimized plan consisted of 23 energy layers ranging from 72.7 MeV to 125.1 MeV. The optimizer automatically determined the weight, spacing, and number of spots per layer, as this is the clinical standard with which patient plans are optimized. The number of spots per layer ranged from 172 to 378, and spot spacing fell between 0.57 cm and 0.72 cm.

With this single‐field uniform dose plan optimized, the beam characteristics remained constant for the remainder of the study; the energy of each layer, layer spacing, spot weight, number of spots and spot placement did not change. Range shifter positional adjustments provided an easy method to vary the air gap, and TPS dose was recalculated for each air gap. Dose calculations were performed for 18 different air gaps, ranging from 0.5 cm to 28 cm. Line dose profiles along the central axis from the phantom surface to a depth of 7 cm were exported from the TPS for each calculated dose distribution.

Monte Carlo calculations were performed in a nearly identical manner to the pencil beam calculations discussed above with the only difference being the dose calculation settings. RayStation 6 allows Monte Carlo calculations to be based on either a defined number of proton histories per spot or a statistical uncertainty threshold, where the statistical uncertainty is defined as the average statistical uncertainty for all voxels with dose larger than 50% of the maximum dose.^5^ Monte Carlo dose calculations were performed at 0.1% uncertainty. All other parameters, including the dose grid, remained constant, and calculations were performed for each of the 18 air gaps. Line dose profiles were exported for all calculated configurations.

### 2.B Experimental setup

The optimized treatment plan was exported to MOSAIQ (Elekta, Sunnyvale, CA, USA) and delivered by the IBA ProteusONE compact‐gantry proton therapy system with a 3.5 cm Lexan range shifter inserted in the retractable snout. Dose was measured with the PTW T23343 Markus chamber (PTW, Freiburg, Germany) embedded in SP34 RW3 solid plate phantom material (IBA‐Dosimetry, Schwarzenbruck, Germany). Dose measurements at a given depth were taken for each air gap by simply moving the range shifter snout to the appropriate position. When all data for one depth were acquired, the chamber was repositioned to the appropriate depth in the phantom, the vertical couch position was adjusted to keep the isocenter position constant, and the measurement process was repeated for all depth/air gap combinations.

### 2.C Data analysis

#### 2.C.1 Absorbed dose calculation

Measurement acquisition occurred over multiple days. To control for daily variations in beam output, a daily output correction factor was calculated and applied to each measurement. This correction factor was calculated from the daily QA central‐axis (CAX) dose of a range‐shifted field as measured by the MatriXX PT ion chamber array (IBA‐Dosimetry, Schwarzenbruck, Germany). Cross‐calibration of the MatriXX PT with the Markus chamber showed the MatriXX PT CAX dose to be accurate within 0.4%. This daily output correction factor (P_DO_) is the ratio of the baseline central‐axis dose determined during machine commissioning to the measured daily CAX dose, and was included in the TRS‐398 absorbed dose calculation.^13^

(1) $$D_{w,Q}=M_{\mathrm{raw}}N_{D,w,Q_{0}}k_{Q,Q_{0}}P_{T,P}P_{\mathrm{ion}}P_{\mathrm{pol}}P_{\mathrm{elec}}P_{\mathrm{DO}}$$

#### 2.C.2 Dosimetric accuracy

All measured values of absorbed dose were tabulated based on air gap and effective depth of measurement. The effective depth of measure is the summation of the amount of solid water above the chamber and the water‐equivalent thickness of the chamber window, which is 3.0 × 10^−3^ cm.^14^

For each air gap configuration, TPS‐calculated dose values at the effective depths of measurement were extracted from the line dose profiles via interpolation. Dosimetric accuracy was then calculated as the ratio of the TPS dose to the measured dose value. These comparative calculations were performed for the TPS pencil beam and Monte Carlo data. Plots of TPS dosimetric accuracy as a function of both depth and air gap were generated.

#### 2.C.3 Clinical analysis

To validate the results of this work and show applicability of the data to clinical cases, shallow dose planes extracted from multiple patient QA plans were analyzed and compared to the findings of this work. Two previously treated PB patient plans, which treated chest wall and paraspinal mets, were calculated to 0.5% statistical uncertainty using the Monte Carlo dose engine. The QA dose planes were extracted from depths of 0.6 cm, 2.6 cm, and 4.6 cm and directly compared to the corresponding PB dose planes. This comparison was performed with OmniPro I'MRT software (IBA‐Dosimetry, Schwarzenbruck, Germany) using various γ‐analysis criteria. This direct comparison of TPS planes was able to quantify the gross difference between PB and MC dose distributions over a large area, rather than the central‐axis‐only data discussed throughout this work.

#### 2.C.4 Validation with commissioning data

To validate the dosimetric accuracy of the Monte Carlo dose algorithm under controlled conditions different than those used in the single‐field beam discussed above, a selection of three commissioning fields were measured and compared to RayStation's PB and Monte Carlo algorithms. Field Comm01 is a 4 × 4 × 4 cm cube uniformly dosed to 200 cGy with isocenter at 20 cm depth and no range shifter. Fields Comm02 and Comm03 are both 10 × 10 × 10 cm cubes uniformly dosed to 200 cGy; Comm02 has isocenter at a depth of 5 cm with a 15 cm range shifter air gap and Comm03 has isocenter at a depth of 10 cm with a 10 cm range shifter air gap.

The three fields used during the initial commissioning of the ProteusONE were measured with the Zebra (IBA‐Dosimetry, Schwarzenbruck, Germany) multi‐layer ion chamber device to obtain depth dose curves of the SOPB's. The commissioning fields were calculated in RayStation 6 with the PB and Monte Carlo dose algorithms and depth dose curves were extracted in the same manner as described previously. Because the Zebra consists of 180 ion chambers spaced approximately 2 mm apart, the measured data were interpolated to match the 0.1 mm resolution of the TPS data. The measured and computed data were compared via gamma analysis.

## 3 RESULTS

### 3.A Surface dose anomaly

This experiment intended to determine the dosimetric accuracy of the TPS for superficial treatments, including surface doses. The thin entrance window of the Markus chamber made it ideal for measuring surface dose. However, all surface measurements (i.e., measurements at a depth of 0.03 mm – the chamber window thickness) showed a significant discrepancy from the TPS calculated doses. This anomaly held true for both the PB and MC dose algorithms, and it showed no significant relationship to air gap.

Surface dose measurements were, on average, 43% higher than the PB TPS dose calculations and 45% higher than the MC TPS calculated dose. This discrepancy does not appear to be a function of the type of dose engine, but rather a fundamental shortcoming of a TPS in general. Whether using an analytical dose model or a Monte Carlo calculation, it appears the TPS will have difficulty in predicting dose values at sub‐millimeter depths. Though the true cause is not fully understood, the authors hypothesize that low‐energy scatter created in the range shifter could be responsible for the elevated dose measurements. A personal communication with a RayStation representative confirmed that the TPS does not model any electron scatter generated in the range shifter. Additionally, the finite nature of voxels and their relatively large size compared to the Markus chamber window thickness leads to significant uncertainty in TPS‐reported dose values at such shallow depths.

All further analysis of dosimetric accuracy excludes these surface doses, as they are clearly significant outliers in otherwise consistent data.

### 3.B Pencil beam algorithm dosimetric accuracy

**Figure 1:**
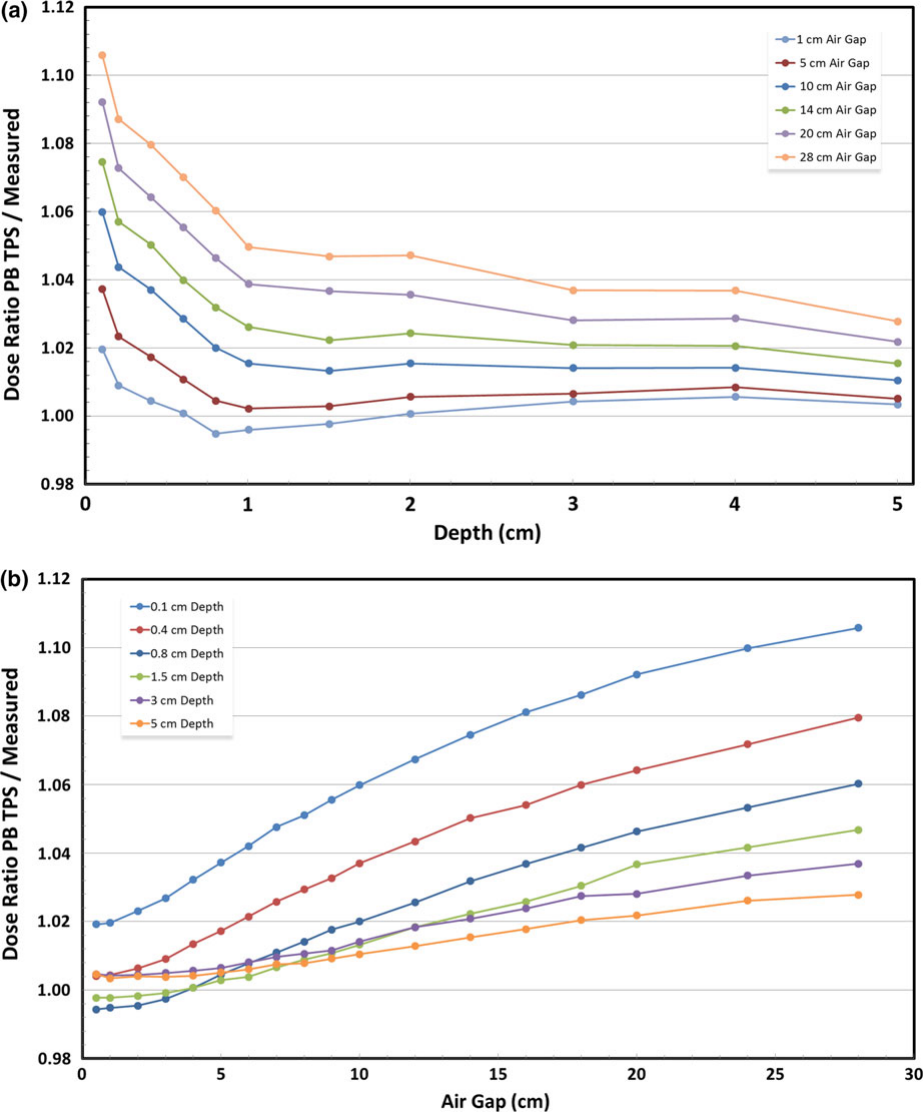
(a) Dosimetric accuracy of a pencil beam TPS relative to measured dose as a function of depth. (b) Dosimetric accuracy of a pencil beam TPS relative to measured dose as a function of air gap.

The dosimetric accuracy of the TPS pencil beam algorithm has a dependence on both depth and air gap, as shown in Figs. 1(a) and 1(b). PB‐calculated TPS doses become more accurate at increasing depths and at decreasing air gaps. When the air gap is relatively small, the TPS accuracy is clinically acceptable (within 3%) at all depths 2 mm and deeper. As the air gap widens, dosimetric accuracy degrades, especially at the shallowest depths. The depth dependence of pencil beam dose algorithm accuracy is strongest in the shallowest 1 cm, eventually stabilizing beyond 3 cm. Table 1 bins the information from Figs. 1(a) and 1(b), while Table 3 shows the complete set of data acquired.

**Table 1:** Dosimetric accuracy of the pencil beam algorithm at various depths and air gaps

Depth (cm)	Air gap (cm)	PB/measured dose	Std dev
<1	<5	1.009	0.010
<1	5–9	1.027	0.014
<1	10–14	1.044	0.016
<1	15–20	1.061	0.017
<1	>20	1.077	0.017
1 ≤ *d* < 3	<5	0.999	0.002
1 ≤ *d* < 3	5–9	1.008	0.004
1 ≤ *d* < 3	10–14	1.020	0.004
1 ≤ *d* < 3	15–20	1.032	0.004
1 ≤ *d* < 3	>20	1.051	0.010
3+	<5	1.005	0.001
3+	5–9	1.009	0.002
3+	10–14	1.016	0.004
3+	15–20	1.024	0.004
3+	>20	1.032	0.005

### 3.C Monte carlo algorithm dosimetric accuracy

**Figure 2:**
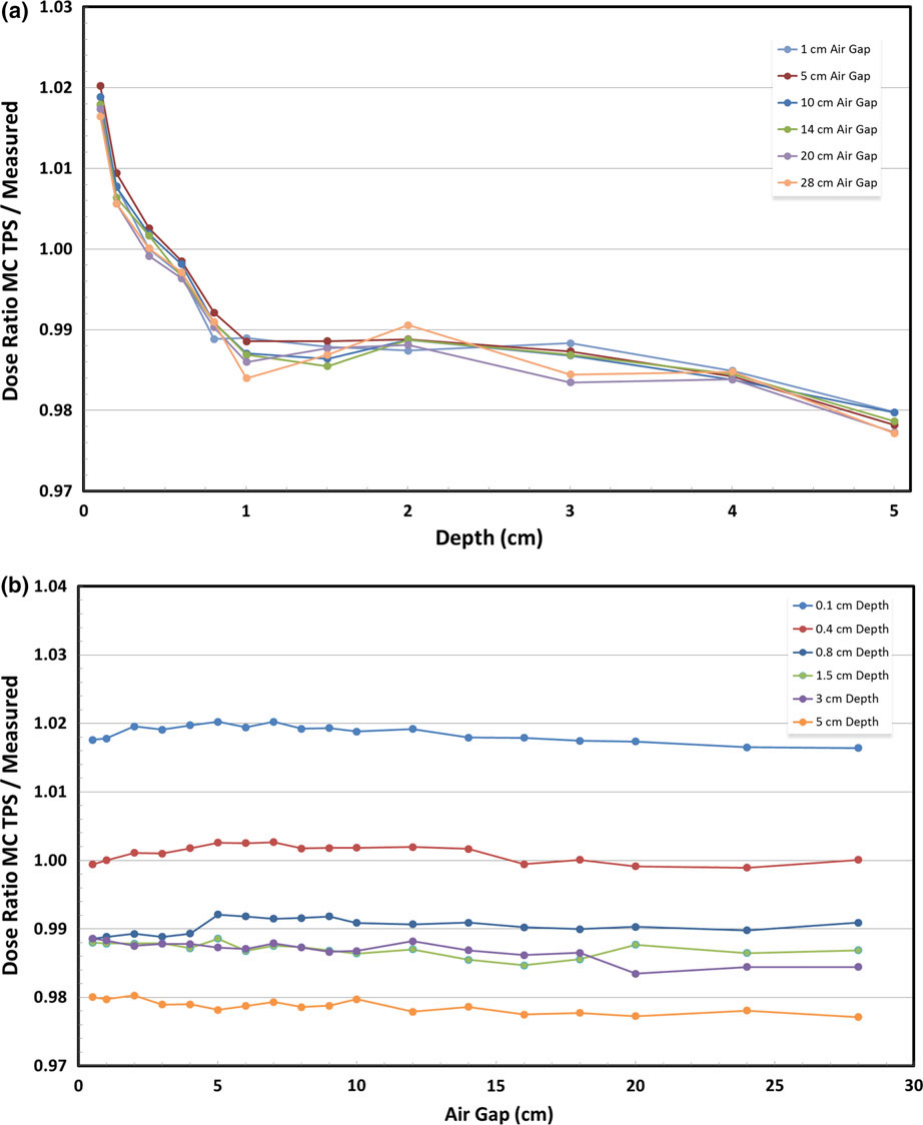
(a) Dosimetric accuracy of a Monte Carlo TPS relative to measured dose as a function of depth. (b) Dosimetric accuracy of a Monte Carlo TPS relative to measured dose as a function of air gap.

Figure 2(a) shows the depth dependence of Monte Carlo TPS dosimetric accuracy. This relationship with depth shares the same general shape as the PB TPS depth dependence, albeit with different magnitude. The greatest change in accuracy occurs over the first centimeter, beyond which the dosimetric accuracy stabilizes. Figure 2(b), however, clearly shows that dosimetric accuracy is not a function of air gap when using a Monte Carlo TPS. Additionally, Monte Carlo underestimated dose at all points deeper than 4 mm. Table 2 displays binned data and Table 4 shows all Monte Carlo data. Figures 1(a), 1(b), 2(a), and 2(b) show a representative subset of the data which allows the observer to understand the trends while minimizing clutter.

**Table 2:** Dosimetric accuracy of the Monte Carlo dose engine at various depths and air gaps

Depth (cm)	Air gap (cm)	MC/measured dose	Std dev
<1	<5	1.003	0.010
<1	5–9	1.004	0.010
<1	10–14	1.003	0.010
<1	15–20	1.002	0.010
<1	>20	1.002	0.009
1 ≤ *d* < 3	<5	0.988	0.001
1 ≤ *d *<* *3	5–9	0.988	0.001
1 ≤ *d *<* *3	10–14	0.987	0.001
1 ≤ *d *<* *3	15–20	0.987	0.001
1 ≤ *d *<* *3	>20	0.990	0.005
3+	<5	0.984	0.004
3+	5–9	0.983	0.004
3+	10–14	0.983	0.004
3+	15–20	0.982	0.004
3+	>20	0.982	0.004

### 3.D Range shifter thickness

Previous works by the authors have reported findings from similar tests, which directly compared the air gap and depth dependences of a 3.5 cm (4.1 cm WET) range shifter to a 6.5 cm (7.4 cm WET) range shifter. On average, the dosimetric error of the thicker range shifter was found to be approximately 50% greater than the thinner range shifter.^15^

### 3.E Clinical validation of data using patient plans

As a clinical test of this data, shallow QA dose planes of a chest wall patient and a patient with paraspinal mets were calculated with both PB and MC dose engines and compared via γ‐analysis. The paraspinal plan was also tested with extended air gaps to illustrate the difference between a well‐planned treatment with air gaps less than 10 cm and a sub‐optimal plan with air gaps greater than 15 cm.

Given the depth and air gap for each field, the expected dose error of the PB and MC calculations were determined by interpolating data in Tables 3 and 4, respectively. The difference of PB and MC dosimetric errors represents the total expected dose difference between datasets. If the data in Tables 3 and 4 is consistent, repeatable, and accurate for central‐axis and off‐axis data in any treatment plan, then one can expect the γ‐analysis comparing the PB and MC dose planes will fail until the percent dose (%D) portion of the γ‐analysis criteria is increased to match the total expected dose difference.

**Table 3:** Ratio of pencil beam TPS dose to measured dose for all depth/air gap combinations measured

Depth (cm)	Air gap (cm)																	
	0.5	1	2	3	4	5	6	7	8	9	10	12	14	16	18	20	24	28
0.00	0.548	0.548	0.550	0.553	0.557	0.560	0.563	0.567	0.570	0.572	0.575	0.579	0.583	0.586	0.590	0.592	0.596	0.599
0.10	1.019	1.020	1.023	1.027	1.032	1.037	1.042	1.048	1.051	1.056	1.060	1.067	1.075	1.081	1.086	1.092	1.100	1.106
0.20	1.009	1.009	1.011	1.014	1.019	1.023	1.028	1.033	1.037	1.041	1.044	1.051	1.057	1.063	1.069	1.073	1.081	1.087
0.40	1.004	1.004	1.006	1.009	1.013	1.017	1.021	1.026	1.029	1.033	1.037	1.043	1.050	1.054	1.060	1.064	1.072	1.080
0.60	1.000	1.001	1.002	1.004	1.007	1.011	1.014	1.018	1.021	1.025	1.029	1.034	1.040	1.045	1.051	1.055	1.063	1.070
0.80	0.994	0.995	0.995	0.997	1.001	1.005	1.008	1.011	1.014	1.018	1.020	1.026	1.032	1.037	1.042	1.046	1.053	1.060
1.00	0.997	0.996	0.997	0.997	0.999	1.002	1.005	1.008	1.010	1.013	1.015	1.021	1.026	1.031	1.034	1.039	1.044	1.050
1.50	0.998	0.998	0.998	0.999	1.001	1.003	1.004	1.007	1.009	1.011	1.013	1.018	1.022	1.026	1.030	1.037	1.042	1.047
2.00	1.001	1.001	1.002	1.003	1.004	1.006	1.007	1.010	1.011	1.014	1.015	1.020	1.024	1.028	1.032	1.036	1.042	1.047
3.00	1.005	1.004	1.004	1.005	1.006	1.007	1.008	1.010	1.011	1.012	1.014	1.018	1.021	1.024	1.027	1.028	1.033	1.037
4.00	1.006	1.006	1.006	1.007	1.007	1.008	1.009	1.010	1.012	1.013	1.014	1.017	1.021	1.024	1.027	1.029	1.033	1.037
5.00	1.005	1.003	1.004	1.004	1.004	1.005	1.006	1.008	1.008	1.009	1.010	1.013	1.015	1.018	1.020	1.022	1.026	1.028

**Table 4:** Ratio of Monte Carlo TPS dose to measured dose for all depth/air gap combinations measured

Depth (cm)	Air gap (cm)																	
	0.5	1	2	3	4	5	6	7	8	9	10	12	14	16	18	20	24	28
0.00	0.546	0.546	0.547	0.548	0.548	0.549	0.549	0.550	0.551	0.550	0.550	0.551	0.550	0.549	0.549	0.549	0.548	0.548
0.10	1.018	1.018	1.020	1.019	1.020	1.020	1.019	1.020	1.019	1.019	1.019	1.019	1.018	1.018	1.017	1.017	1.017	1.016
0.20	1.008	1.008	1.009	1.008	1.009	1.009	1.009	1.009	1.009	1.009	1.008	1.008	1.006	1.007	1.007	1.006	1.005	1.006
0.40	0.999	1.000	1.001	1.001	1.002	1.003	1.003	1.003	1.002	1.002	1.002	1.002	1.002	0.999	1.000	0.999	0.999	1.000
0.60	0.996	0.997	0.997	0.997	0.997	0.999	0.998	0.998	0.998	0.998	0.998	0.997	0.997	0.996	0.996	0.996	0.996	0.997
0.80	0.989	0.989	0.989	0.989	0.989	0.992	0.992	0.991	0.992	0.992	0.991	0.991	0.991	0.990	0.990	0.990	0.990	0.991
1.00	0.989	0.989	0.989	0.988	0.988	0.989	0.989	0.989	0.988	0.988	0.987	0.987	0.987	0.986	0.985	0.986	0.985	0.984
1.50	0.988	0.988	0.988	0.988	0.987	0.989	0.987	0.988	0.987	0.987	0.986	0.987	0.985	0.985	0.986	0.988	0.986	0.987
2.00	0.987	0.987	0.988	0.988	0.989	0.989	0.988	0.989	0.989	0.989	0.989	0.989	0.989	0.988	0.988	0.988	0.989	0.991
3.00	0.989	0.988	0.988	0.988	0.988	0.987	0.987	0.988	0.987	0.987	0.987	0.988	0.987	0.986	0.986	0.983	0.984	0.984
4.00	0.986	0.985	0.986	0.985	0.985	0.984	0.984	0.984	0.985	0.985	0.984	0.983	0.984	0.985	0.984	0.984	0.984	0.985
5.00	0.980	0.980	0.980	0.979	0.979	0.978	0.979	0.979	0.979	0.979	0.980	0.978	0.979	0.978	0.978	0.977	0.978	0.977

Tables 5 and 6 show the depth, air gap, estimated errors, and total expected dose difference for Field 01 LAO and Field 02 LAO of the chest wall patient and Field 03 RPO and Field 04 RPO of the paraspinal mets patient, respectively. Table 7 shows the corresponding data for the paraspinal patient when the air gap has been extended an additional 10 cm for each field – these fields have been identified as Field 03a RPO and Field 04a RPO.

**Table 5:** Depth, air gap, estimated errors, and total expected dose difference for chest wall fields using PB and MC dose engines

Field	Dose engine	Depth (cm)	Air gap (cm)	Estimated error	% Dose difference
01 LAO	PB	0.6	15.36	1.044	4.8
01 LAO	MC	0.6	15.36	0.996	
01 LAO	PB	2.6	15.36	1.025	3.8
01 LAO	MC	2.6	15.36	0.987	
01 LAO	PB	4.6	15.36	1.019	3.8
01 LAO	MC	4.6	15.36	0.981	
02 LAO	PB	0.6	24.35	1.068	7.1
02 LAO	MC	0.6	24.35	0.997	
02 LAO	PB	2.6	24.35	1.041	5.3
02 LAO	MC	2.6	24.35	0.988	
02 LAO	PB	4.6	24.35	1.031	5.1
02 LAO	MC	4.6	24.35	0.980	

**Table 6:** Depth, air gap, estimated errors, and total expected dose difference for paraspinal fields using PB and MC dose engines

Field	Dose engine	Depth (cm)	Air gap (cm)	Estimated error	% Dose difference
03 RPO	PB	0.6	7.59	1.0200	2.2
03 RPO	MC	0.6	7.59	0.998	
03 RPO	PB	2.6	7.59	1.010	2.2
03 RPO	MC	2.6	7.59	0.988	
03 RPO	PB	4.6	7.59	1.009	2.8
03 RPO	MC	4.6	7.59	0.981	
04 RPO	PB	0.6	6.62	1.017	1.9
04 RPO	MC	0.6	6.62	0.998	
04 RPO	PB	2.6	6.62	1.009	2.1
04 RPO	MC	2.6	6.62	0.988	
04 RPO	PB	4.6	6.62	1.008	2.7
04 RPO	MC	4.6	6.62	0.981	

**Table 7:** Depth, extended air gap, estimated errors, and total expected dose difference for paraspinal fields using PB and MC dose engines

Field	Dose engine	Depth (cm)	Air gap (cm)	Estimated error	% Dose difference
03a RPO	PB	0.6	17.59	1.050	5.4
03a RPO	MC	0.6	17.59	0.996	
03a RPO	PB	2.6	17.59	1.029	4.2
03a RPO	MC	2.6	17.59	0.987	
03a RPO	PB	4.6	17.59	1.022	4.2
03a RPO	MC	4.6	17.59	0.980	
04a RPO	PB	0.6	16.62	1.047	5.1
04a RPO	MC	0.6	16.62	0.996	
04a RPO	PB	2.6	16.62	1.027	4.0
04a RPO	MC	2.6	16.62	0.987	
04a RPO	PB	4.6	16.62	1.021	4.1
04a RPO	MC	4.6	16.62	0.980	

A series of γ‐analyses were performed for each field, with the %D criteria incrementally increased until nearly all points (>99%) passed, as shown in Table 8, 9, and 10. Field 01 LAO, measured at a depth of 0.6 cm with an air gap of 15.4 cm, had an expected dose difference of 4.7% between the PB and MC dose calculations and required γ‐analysis criteria of 5%/1 mm to pass. Field 02 LAO, with a larger air gap of 24.4 cm, had a greater expected dose difference of 7.1% at a depth of 0.6 cm. The γ‐analysis of Field 02 LAO required analysis criteria of 7%/1 mm in order to pass. Similar results are shown for the 2.6 cm and 4.6 cm depths, as well as Fields 03 RPO, 04 RPO, 03a RPO, and 04a RPO.

**Table 8:** γ‐analysis pass rates comparing chest wall PB to MC fields with varying γ‐analysis criteria

Field	Depth (cm)	Air gap (cm)	Expected %D	γ‐Analysis pass rate (%)					
				2%/1 mm	3%/1 mm	4%/1 mm	5%/1 mm	6%/1 mm	7%/1 mm
01 LAO	0.6	15.36	4.8	52.04	74.66	93.77	99.59	–	–
01 LAO	2.6	15.36	3.8	66.82	92.40	99.20	–	–	–
01 LAO	4.6	15.36	3.8	71.40	92.27	99.72	–	–	–
02 LAO	0.6	24.35	7.1	37.50	50.90	62.29	93.10	97.86	99.96
02 LAO	2.6	24.35	5.3	51.95	68.21	86.90	99.19	–	–
02 LAO	4.6	24.35	5.1	63.70	79.80	95.18	99.82	–	–

**Table 9:** γ‐analysis pass rates comparing paraspinal PB to MC fields with varying γ‐analysis criteria

Field	Depth (cm)	Air gap (cm)	Expected %D	γ‐Analysis pass rate (%)		
				2%/1 mm	3%/1 mm	4%/1 mm
03 RPO	0.6	7.59	2.2	79.71	97.42	99.89
03 RPO	2.6	7.59	2.2	96.13	99.74	–
03 RPO	4.6	7.59	2.8	98.26	99.95	–
04 RPO	0.6	6.62	1.9	92.93	99.89	–
04 RPO	2.6	6.62	2.1	99.11	–	–
04 RPO	4.6	6.62	2.7	95.75	99.96	–

**Table 10:** γ‐analysis pass rates comparing extended air gap paraspinal PB to MC fields with varying γ‐analysis criteria

Field	Depth (cm)	Air gap (cm)	Expected %D	γ‐Analysis pass rate (%)				
				2%/1 mm	3%/1 mm	4%/1 mm	5%/1 mm	6%/1 mm
03a RPO	0.6	17.59	5.4	32.17	50.52	73.84	95.67	99.19
03a RPO	2.6	17.59	4.2	57.10	93.10	99.44	–	–
03a RPO	4.6	17.59	4.2	82.22	98.83	99.99	–	–
04a RPO	0.6	16.62	5.1	47.53	60.38	78.90	99.52	–
04a RPO	2.6	16.62	4.0	65.97	95.78	99.98	–	–
04a RPO	4.6	16.62	4.1	76.79	96.27	99.98	–	–

All fields, with the exception of Field 03 RPO at a depth of 0.6 cm, have very good agreement between expected dose difference and γ‐analysis dose difference required for a passing QA test. This confirms the applicability of the data collected in this work with respect to other clinical patient treatment plans.

### 3.F Validation with commissioning data

**Figure 3:**
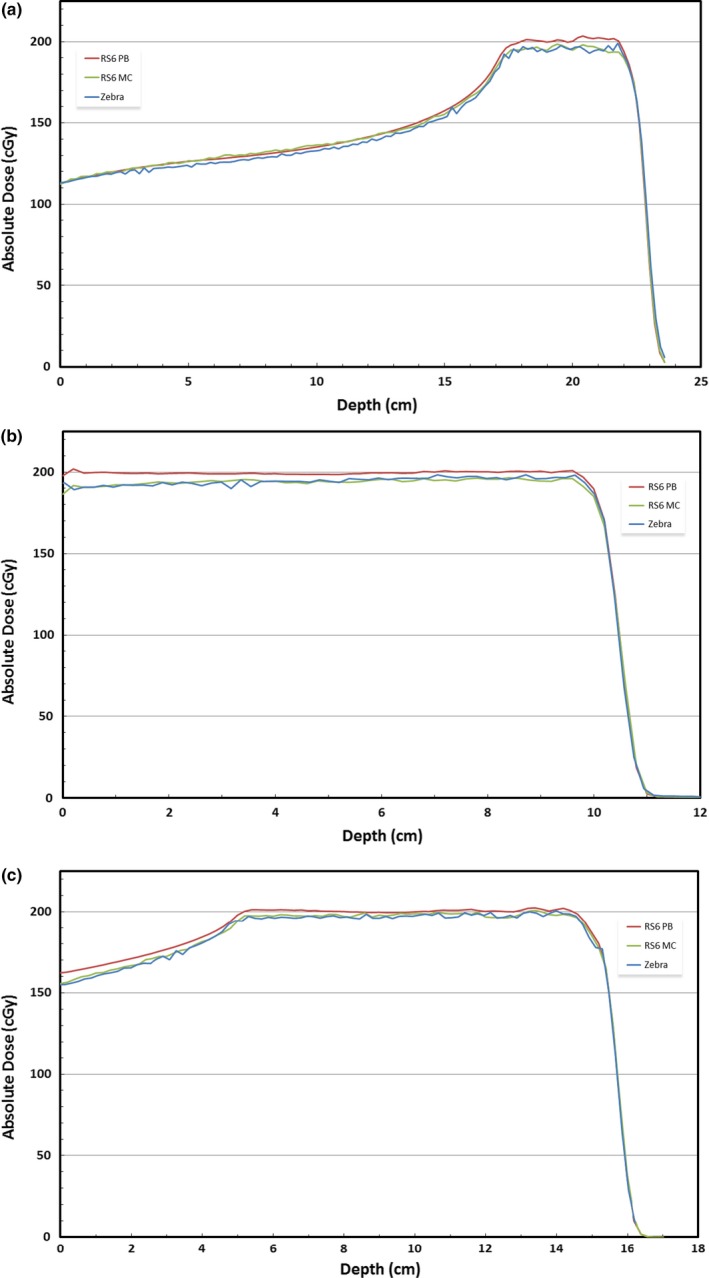
Depth dose curves calculated with RayStation 6 pencil‐beam and Monte Carlo dose algorithms and measured with Zebra MLIC for three commissioning fields: (a) Comm01; (b) Comm02; (c) Comm03.

Because the majority of data collected for this work were based on a single treatment field, three additional fields of varying energy and field size were modeled in the TPS and compared against measured data. Figure 3 shows three separate plots – one for each treatment field – which depict the depth dose curves as calculated by RayStation 6 pencil‐beam and Monte Carlo dose algorithms and as measured by the Zebra multi‐layer ion chamber (MLIC). The plots clearly show consistent agreement between the Monte Carlo calculation and the measured data while the pencil‐beam data differs rather significantly.

A γ‐analysis was performed for each field, comparing both the MC and PB calculated data with the Zebra measurements. Using a 3%/3 mm γ‐analysis criteria, the Monte Carlo fields had passing rates of 100%, 99.75%, and 100% for fields Comm01, Comm02, and Comm03, respectively. The PB data had corresponding passing rates of 93.81%, 76.47%, and 93.48%.

## 4 DISCUSSION

As a direct result of this research, Willis‐Knighton Cancer Center has implemented multiple changes to the way proton therapy patients are treated. Because the ProteusONE system is capable of producing protons with energy as low as 70 MeV, which have a range of approximately 4.1 cm, there is no practical reason that a range shifter significantly thicker than 4 cm would be necessary. Even though WKCC commissioned both the 4.1 cm and 7.4 cm WET range shifters, given the fact that a 7.4 cm range shifter has, on average, 50% greater TPS dosimetric error than a 4.1 cm range shifter, only the 4.1 cm range shifter will be used.

In addition to minimizing range shifter thickness, WKCC adopted a policy to keep the air gap less than 10 cm when patient setup and machine geometry allow. Based on patient arm position, gantry angle, and range shifter dimensions, certain patients may not accommodate such small air gaps. For example, when a chest wall patient places her arms over her head, her elbows may prevent the range shifter from extending as far as necessary to achieve an appropriate air gap. In such cases, the patient is brought in prior to treatment to determine exactly how far the range shifter can extend without colliding with the patient. If a 10 cm air gap cannot be achieved, all efforts are made to minimize the air gap as much as possible.

Proton facilities treating superficially may consider performing similar measurements to understand the discrepancy between TPS and measured dose. WKCC collected a small set of similar data during machine and TPS commissioning, but it was not until superficial treatments became commonplace that the true magnitude of TPS dosimetric inaccuracy became clear. Having a comprehensive data set of TPS dosimetric accuracy for superficial treatments ensures that the physicians, dosimetrists, and physicists are all aware of this issue and can make informed decisions when treating patients superficially.

## 5 CONCLUSION

For the first time, this study comprehensively quantifies TPS dosimetric accuracy of range‐shifted proton fields as a function of depth, air gap, and range shifter thickness. When pencil beam dose algorithms are used to create superficial PBS treatments, the air gap should be reduced as much as patient setup allows, and range shifter thickness should be minimized to correspond with the range of the machine's minimum energy. Poor modeling of secondary proton scatter generated in the range shifter, also known as the nuclear halo effect, is the main contributor to TPS dose overestimation.^5^ As mentioned by RayStation and as confirmed by this study, implementation of a Monte Carlo dose engine has helped mitigate this error.

## CONFLICT OF INTEREST

The authors have no conflicts of interest to disclose.
